# Bibliographical

**Published:** 1868-01

**Authors:** 


### BIBLIOGRAPHICAL.

REGISTER PAPERS: *A collection of Chemical Essays in reference to Dental Surgery.* By GEO. WATT, M. D., D. D. S., *Professor of Pathology, late Professor of Chemistry and Metallurgy in the Ohio College of Dental Surgery, &c., &c.*

To those who have been constant readers of the DENTAL REGISTER for years past, but little need be said in reference to the essays constituting this work, further than the fact that they are now presented to the profession in a beautifully executed volume. These papers were written at various times during the last ten years, each one under the pressure of a manifestly urgent want in the profession. They were not written, as is too often the case, merely to make a book; but because the demand was so great, that it was a matter of necessity that they be written; and each one as it appeared filled a space—and thus far most completely—that had not been occupied before.

Perhaps in all Dental literature there is nothing that has met with more unqualified approbation at the hands of the Profession than some of these papers. As examples of these we may refer to the articles on the "*Chemistry of the Mouth*, "*Dental Caries*," "*The action of Topical Remedies*," &c., &c. One great value attached to these articles, in addition to their correctness and fullness, is their essentially practical character. The principles are such as are involved in every day practice.

We have frequently been called upon to furnish a number of the REGISTER containing some one of these Essays, and usually, much to the regret of the applicant we have not been able to do it: but we are glad to say that the way is now open for all to avail themselves of the whole series. It is published by S. S. White, and can be obtained at the Dental Depots, and we presume at the principal book stores. Price $2.00. T.

